# Supplementary material for: Immortalization of Salivary Gland Epithelial Cells of Xerostomic Patients: Establishment and Characterization of Novel Cell Lines
Source: J Clin Med. 2020 Nov 25;9(12):3820. doi: 10.3390/jcm9123820 (PMC7768371; doi:10.3390/jcm9123820)
Supplement: Supplementary file 1 [file jcm-09-03820-s001.zip › Supplementary Figure S1 iSGEC 11-24-20 .docx]

**Figure S1.** Semi-qRT-PCR analysis of SV40Lt expression in late passaged iSGECs


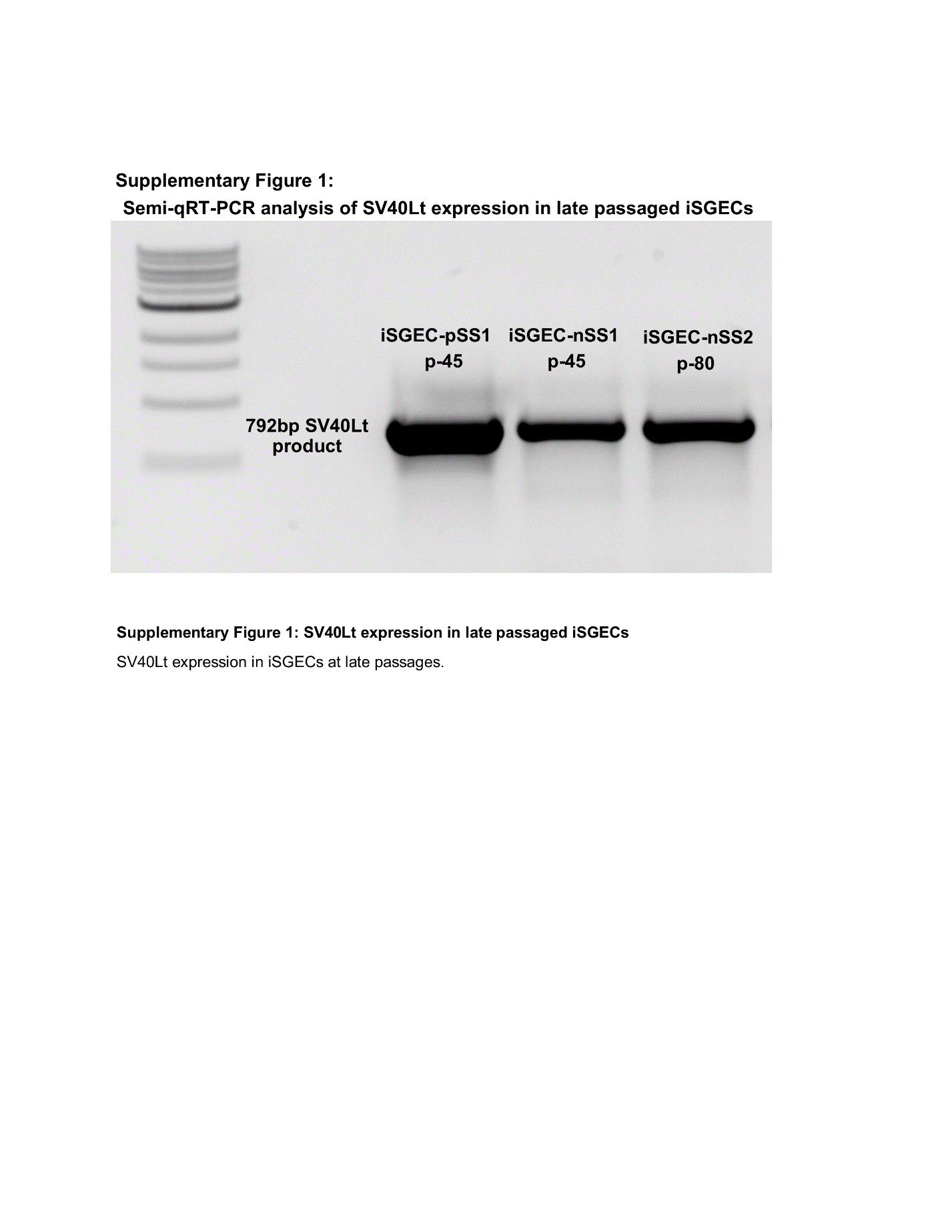


**Legend.**

SV40Lt expression in the three iSGEC lines (pSS1, nSS1, and nSS2) at late passages p-45 and p-80.
