# Supplementary material for: Immortalization of Salivary Gland Epithelial Cells of Xerostomic Patients: Establishment and Characterization of Novel Cell Lines
Source: J Clin Med. 2020 Nov 25;9(12):3820. doi: 10.3390/jcm9123820 (PMC7768371; doi:10.3390/jcm9123820)
Supplement: Supplementary file 1 [file jcm-09-03820-s001.zip › Supplementary Figure S2 iSGEC11-24-20 .docx]

**Figure S2.** Phase contrast microscopy images of generated iSGEC lines

**A. p-80 single cell colonies**


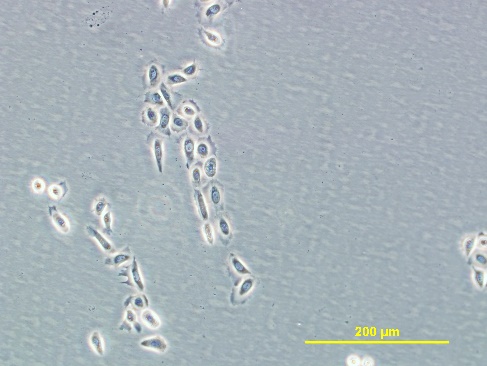

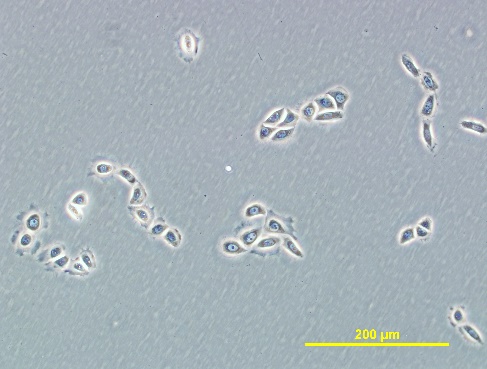

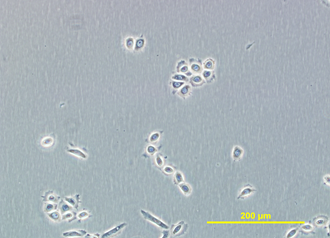


**A**

**B-G. iSGEC-pSS1 and iSGEC-nSS1 spheroids**

**iSGEC-pSS1 (p14)**

**24 hrs (40x)**


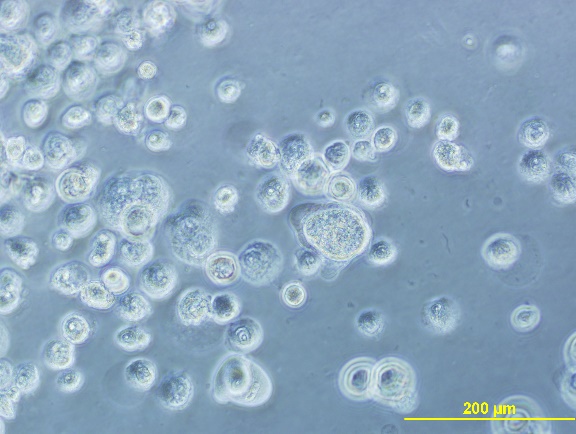

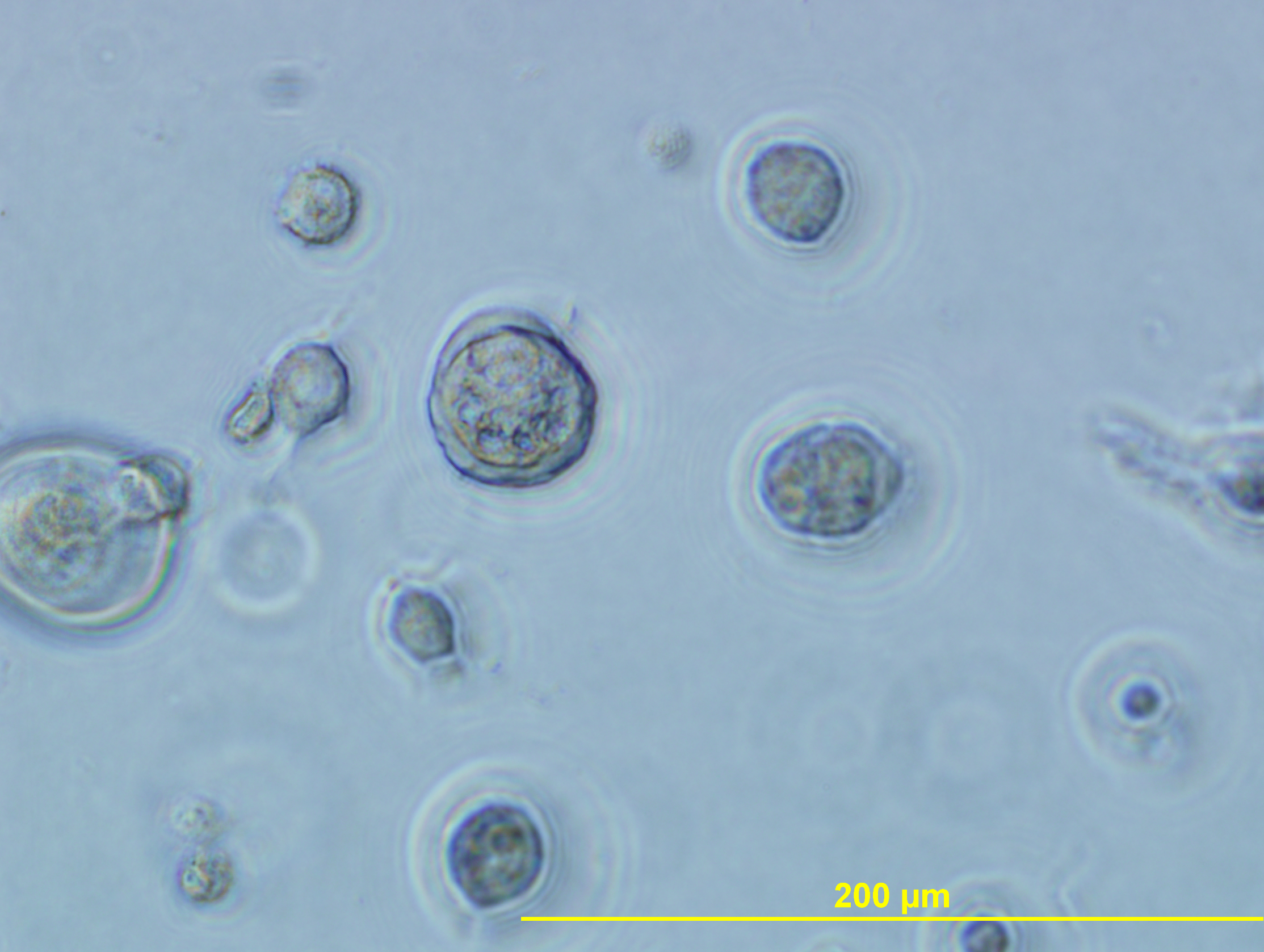

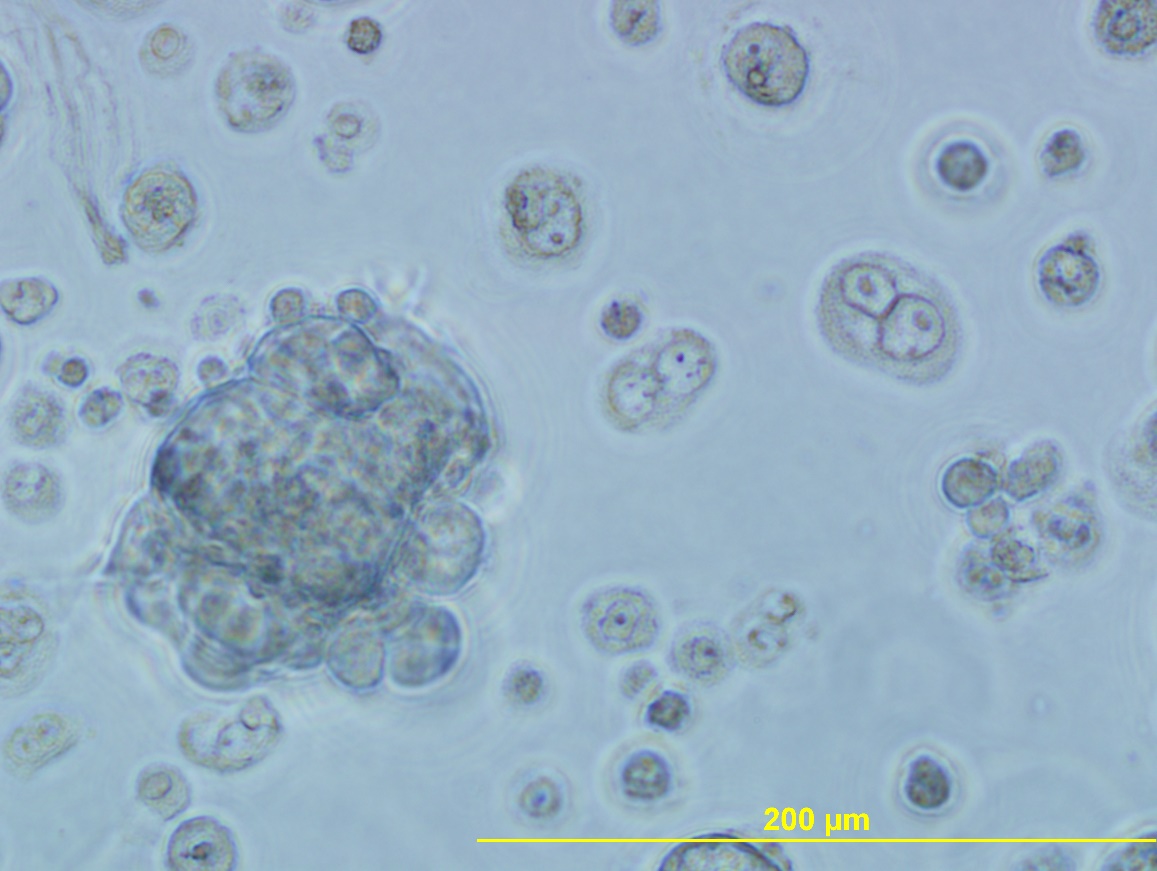


**iSGEC-pSS1 (p14)**

**24 hrs (20x)**

**iSGEC-pSS1 (p14)**

**24 hrs (40x)**


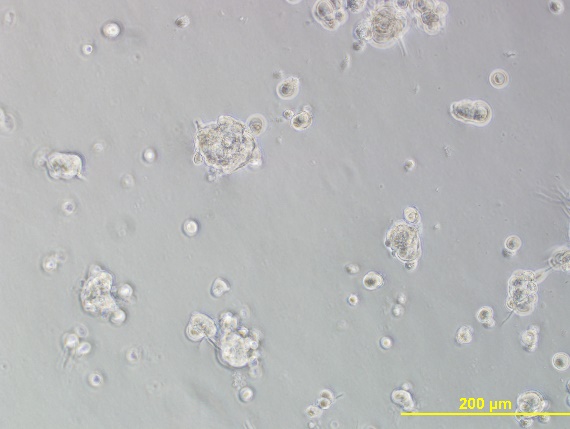


**iSGEC-nSS1 (p14)**

**24 hrs (20x)**


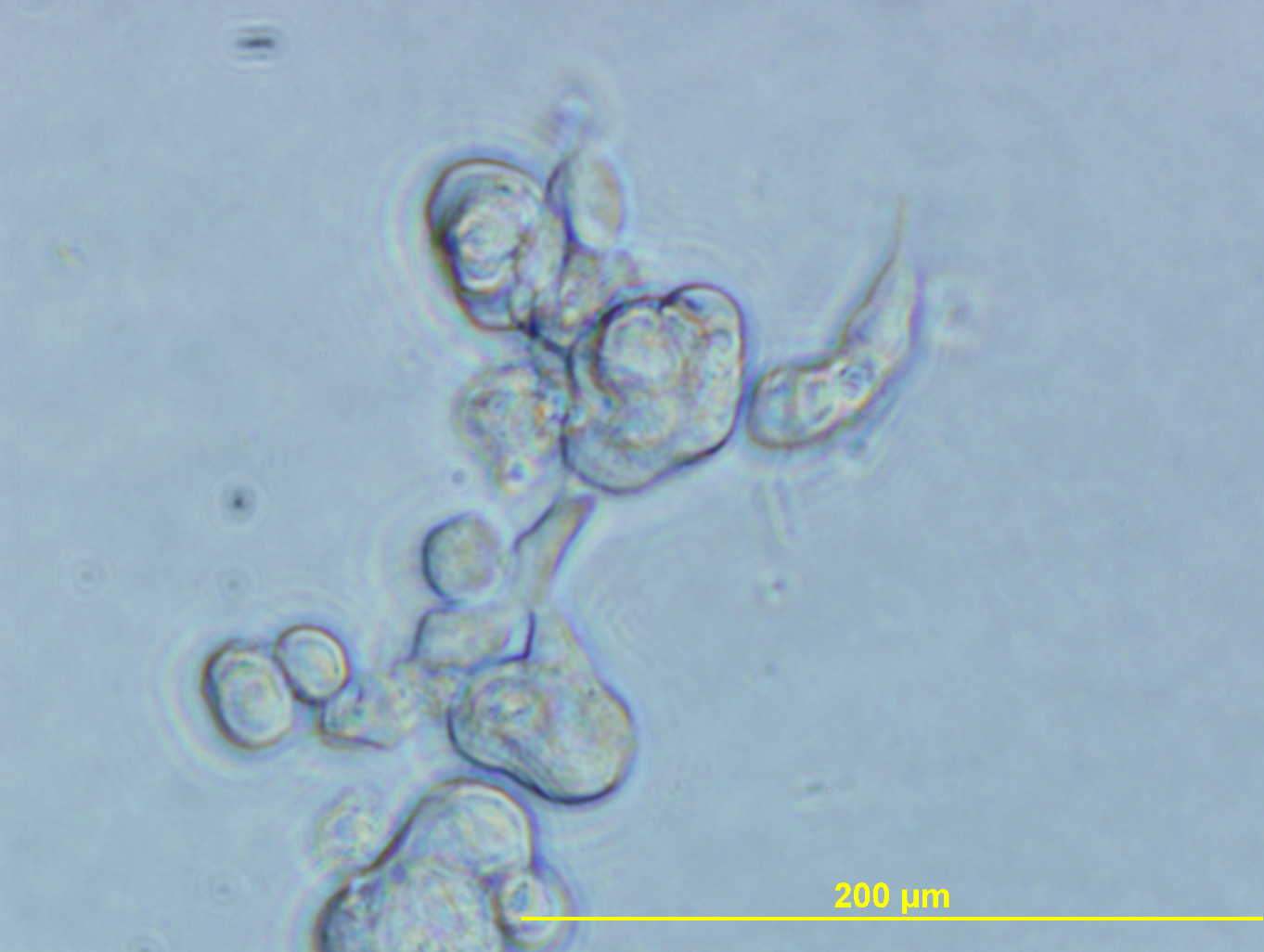


**iSGEC-nSS1 (p14)**

**24 hrs (40x)**


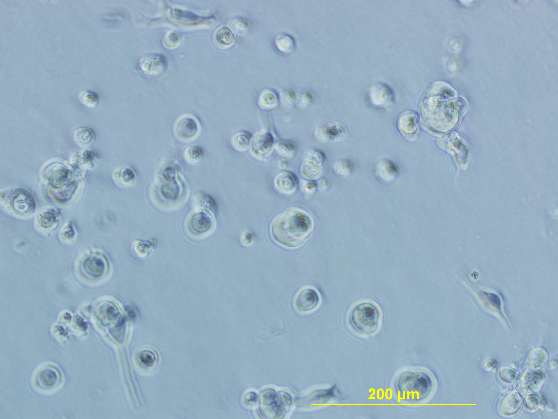


**iSGEC-nSS1 (p14)**

**24 hrs (20x)**

**B**

**E**

**C**

**D**

**F**

**G**

**Legend.**

**A**) Single cell suspensions of iSGEC-nSS2 p-80 were plated on tissue culture treated plastic 12-well plates and grown for a minimum of 5-days before photographing. Linear shaped colonies formed from either filiform-appearing or cuboidal appearing cells. Circular shaped colonies would mainly arise from polygonal/ cuboidal appearing cells. Scale bar represents 200µm (20x). **B-G**) iSGEC-pSS1 and iSGEC-nSS1 were cultured on matrigel for 24 hours before photographing. iSGEC-pSS1 (**B-D**) formed spheroids with apparent differentiation into acinar-like structures (**C**) and myoepithelial-like structures (**D**). iSGEC-nSS1 formed spheroids (**E-F**) like other iSGECs, however, some spheroids exhibited tubular-like protrusions (**G**) resembling ductal formation. Scale bar represents 200µm (**B, E, F**) (20x) and 50µm (**C, D, G**) (40x).
