# Supplementary material for: Immortalization of Salivary Gland Epithelial Cells of Xerostomic Patients: Establishment and Characterization of Novel Cell Lines
Source: J Clin Med. 2020 Nov 25;9(12):3820. doi: 10.3390/jcm9123820 (PMC7768371; doi:10.3390/jcm9123820)
Supplement: Supplementary file 1 [file jcm-09-03820-s001.zip › Supplementary Figure S3 iSGEC 11-24-20 .docx]

**Figure S3.** Expression of characterization markers in iSGEC-nSS2 cells by ICC


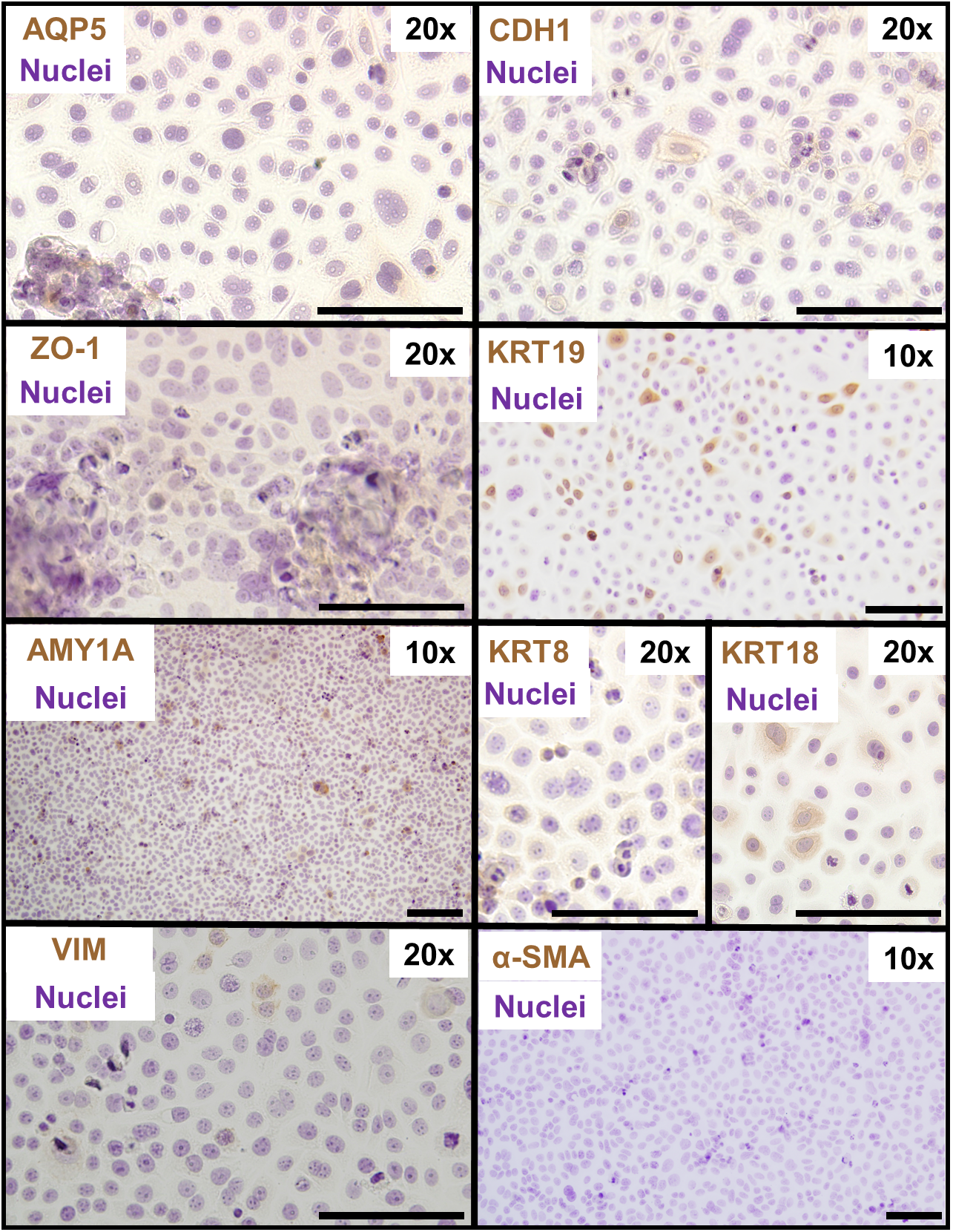


**Legend.**

iSGEC-nSS2 cultures at p-14 were cultured on type-1 collagen coated glass coverslips and incubated with the antibodies . Protein targets (**brown**) are listed per each respective section. Nuclei (**purple**) were counterstained using Hematoxylin. Among proteins indicated, α-SMA was not detected in culture. The scale bar represents 200µm.
