# Supplementary material for: Immortalization of Salivary Gland Epithelial Cells of Xerostomic Patients: Establishment and Characterization of Novel Cell Lines
Source: J Clin Med. 2020 Nov 25;9(12):3820. doi: 10.3390/jcm9123820 (PMC7768371; doi:10.3390/jcm9123820)
Supplement: Supplementary file 1 [file jcm-09-03820-s001.zip › Supplementary Tables 11-24-20.docx]

**Table S1.** RT-PCR Primers

| **Target** | **Primer-F** | **Primer-R** |
| --- | --- | --- |
| ***Acinar cell markers*** |  |  |
| **AMY1A** | CCTTCTGGGATGCTAGGCTG | ATCTTGGCCAACGGTAGCTT |
| **AQP5** | GCTCACTGGGTTTTCTGGGTA | CCTCGTCAGGCTCATACGTG |
| **ANO1** | CTGTCTTCATGGCCCTCTGG | TGACAGCCTCCTCTTCCTCT |
| **ORAI1** | GATGAGCCTCAACGAGCACT | ATTGCCACCATGGCGAAGC |
| **STIM1** | GGGATCTCAGAGGGATTTGACC | TCATGGCATTGAGAGCCTCG |
| **STIM2** | CAGTGACCGGAGTCACAGAC | CAGTTATGAGGTGGGCGTGT |
| **SLC12A2** | TTGGGCCCGATTTTCGAGAG | GGCTGACTGAGGATCTGCAAG |
| **TRPC1** | GTGATGGCGCTGAAGGATGT | ATAGTCACCCTTGTCGCACG |
| **CST3** | AGCCACATCTGAAAAGGAAAGC | GGGTGGGAGGTGTGCATAAG |
| ***Epithelial*** |  |  |
| **ZO-1** | CGGTCCTCTGAGCCTGTAAG | GGATCTACATGCGACGACAA |
| **CLDN1** | CCCAGTCAATGCCAGGTACG | CAAAGTAGGGCACCTCCCAG |
| **CDH1** | CGCATTGCCACATACACTCT | TTGGCTGAGGATGGTGTAAG |
| **KRT19** | CCTCCCGAGATTACAACCACT | GGCGAGCATTGTCAATCTGT |
| ***Myoepithelial*** |  |  |
| **α-SMA** | CTATGCCTCTGGACGCACAACT | CAGATCCAGACGCATGATGGCA |
| ***Mesenchymal*** |  |  |
| **VIM** | CGGGAGAAATTGCAGGAGGA | AAGGTCAAGACGTGCCAGAG |
| ***Progenitor*** |  |  |
| **KRT5** | TTGGACCAGTCAACATCTCTGT | CACTGCTACCTCCGGCAAGA |
| **Nanog** | AGTCCCAAAGGCAAACAACCCACTTC | TGCTGGAGGCTGAGGTATTTCTGTCTC |
| ***Housekeeping*** |  |  |
| **GAPDH** | AGGGCTGCTTTTAACTCTGGT | CCCCACTTGATTTTGGAGGGA |
| ***Immortalization*** |  |  |
| **SV-40** | AGCCTGTAGAACCAAACATT | CTGCTGACTCTCAACATTCT |

| **Gene Symbol** | **Protein Target** | **Dilution** | | **Company** | **Cat#** |
| --- | --- | --- | --- | --- | --- |
|  |  | **ICC** | **Western** |  |  |
| KRT8 | Cytokeratin-8 | 1:250 |  | SCBT | sc-8020 |
| KRT18 | Cytokeratin-18 | 1:250 |  | SCBT | sc-6259 |
| ACTA2 | α smooth muscle actin (α-SMA) | 1:250 |  | SCBT | sc-53015 |
| AQP5 | Aquaporin 5 (AQP5) | 1:100 | 1:300 | SCBT | sc-514022 |
| AMY1A | α-amylase | 1:250 | 1:400 | SCBT | sc-46657 |
| TJP1 | Zona-Occudin- 1 (ZO-1) | 1:100 | 1:300 | SCBT | sc-33725 |
| KRT19 | Cytokeratin-19 | 1:250 |  | SCBT | sc-6278 |
| MKI67 | Ki-67 | 1:250 |  | SCBT | sc-23900 |
| VIM | Vimentin | 1:250 |  | SCBT | sc-6260 |
| CDH1 | E-Cadherin | 1:200 |  | SCBT | sc-8426 |
| VCL | Vinculin |  | 1:1000 | SCBT | sc-73614 |
|  | (Secondary) anti-mouse IgG-HRP | 1:50-1:250 |  | SCBT | sc-516102 |
|  | (Secondary) anti-mouse IgG-HRP |  | 1:1000-3000 | Cell Signaling Tech. | #7076 |
|  | (Secondary) Goat anti-Mouse IgG-Alexa Fluor 594 | 1:250-500 |  | Invitrogen | A11005 |

**Table S2.** Antibodies and dilutions used in ICC and western blots

**Table S2 Footnote.**

Antibodies and concentrations used for western blots and immunocytochemistry (ICC) experiments using horseradish peroxidase (HRP) and immunofluorescence (IF) based detection.
